# Wellbeing and Arthritis Incidence: the Survey of Health, Ageing and Retirement in Europe

**DOI:** 10.1007/s12160-015-9764-6

**Published:** 2016-01-14

**Authors:** Judith A. Okely, Cyrus Cooper, Catharine R. Gale

**Affiliations:** Centre for Cognitive Ageing and Cognitive Epidemiology, Department of Psychology, University of Edinburgh, 7 George Square, Edinburgh, EH8 9JZ UK; MRC Lifecourse Epidemiology Unit, University of Southampton, Southampton, UK

**Keywords:** Wellbeing, Arthritis, CASP-12, Ageing, Longitudinal study

## Abstract

**Background:**

A number of studies provide evidence for an association between psychosocial factors and risk of incident arthritis. Current evidence is largely limited to the examination of negative factors such as perceived stress, but positive factors such as subjective wellbeing may also play a role.

**Purpose:**

The purpose of the current study was to investigate whether people with higher subjective wellbeing have a lower risk of developing arthritis.

**Methods:**

We used Cox proportional hazards regression to examine the prospective relationship between wellbeing (measured using the CASP-12) and incidence of arthritis over a 9-year period. The sample consisted of 13,594 participants aged ≥50 years from the Survey of Health, Ageing and Retirement in Europe.

**Results:**

There was a significant association between greater wellbeing and reduced incident arthritis that was stronger at younger ages. In sex-adjusted analyses, for a standard deviation increase in CASP-12 score, the hazard ratios (95 % confidence intervals) for incident arthritis in people aged <65 and ≥65 years were 0.73 (0.69–0.77) and 0.80 (0.77–0.85), respectively. After further adjustment for other established risk factors, these associations were attenuated but remained significant in both age groups: the fully adjusted hazard ratios were 0.82 (0.77–0.87) and 0.88 (0.82–0.95), respectively.

**Conclusions:**

These results provide evidence for an association between greater wellbeing and reduced risk of incident arthritis and, more generally, support the theory that psychosocial factors are implicated in the aetiology of this disease. Future research needs to delineate the mechanisms underlying the association between wellbeing and arthritis risk.

**Electronic supplementary material:**

The online version of this article (doi:10.1007/s12160-015-9764-6) contains supplementary material, which is available to authorized users.

## Introduction

Arthritis is a significant cause of disability, chronic pain and reduced quality of life, particularly for older adults. Prevalence rates are close to 50 % in the middle aged and older population [1, 2]. Interventions designed to reduce the incidence of arthritis have traditionally adopted the strategy of reducing the prevalence of factors associated with increased risk of two of its major forms, rheumatoid arthritis and osteoarthritis. Risk factors include obesity [3] and joint trauma [4], in the case of osteoarthritis, and smoking [5] in the case of rheumatoid arthritis. Recently, it has been suggested that psychosocial factors may present an additional point of intervention [6].

A number of studies provide evidence for an association between psychosocial factors and risk of incident arthritis. Harris et al. [6] found a significant association between perceived stress and arthritis incidence in a cohort of Australian women (mean age = 52.5 years, SD = 1.5), such that women that experienced high levels of stress were 2.4 times more likely to have developed arthritis 3 years later. Cross-sectional research into arthritis and psychiatric disorders has documented significantly higher prevalence of psychiatric disorders among people diagnosed with arthritis compared with people diagnosed with other chronic conditions such as chronic obstructive pulmonary disease [7]. More recently, longitudinal research which aimed to elucidate the direction of this association has demonstrated that a diagnosis of arthritis increases risk of onset of psychiatric disorders but found no indication that psychiatric disorders led to an increased risk of arthritis incidence [8]. Additional evidence for the role of psychosocial factors in the aetiology of arthritis is provided by a number of studies reporting an association between traumatic experience in childhood and increased arthritis risk in adulthood [9–11].

Although these studies provide some indication that psychosocial factors may influence arthritis risk, evidence for this is largely limited to the examination of negative factors. Positive factors such as subjective wellbeing may also play a role. Numerous studies have reported an association between aspects of wellbeing and health outcomes including all-cause mortality [12] and incidence of some chronic diseases [13–16]. Significantly, the association between wellbeing and physical health or longevity appears to be partially independent of negative emotion as the association remains significant after controlling for negative psychosocial factors such as depression [12, 16, 17].

In a previous study, we examined the association between subjective wellbeing and arthritis risk for the first time [18]. Using a sample of 8182 people aged 50 years and over from the English Longitudinal Study of Ageing, we investigated the association between wellbeing and the incidence of a range of chronic diseases including arthritis over an 8-year period. After adjusting for established risk factors, we found that a standard deviation (SD) increase in wellbeing score was associated with an 11 % decrease in arthritis risk. Our findings suggest that older people with higher levels of wellbeing have a reduced risk of incident arthritis; however, due to a lack of previous research in this area, further investigation is warranted.

The primary aim of the current study was to assess the association between wellbeing and arthritis risk in a nationally representative European sample of community-dwelling individuals aged 50 years and over.

## Methods

### Study Population

The Survey of Health, Ageing and Retirement in Europe is a multi-national prospective cohort study of people aged 50 years and over [19]. Based on probability samples, the Survey of Health, Ageing and Retirement in Europe is designed to be representative of the older community-dwelling population in 20 European countries and Israel. In the first wave of the study (2004/2005), 30, 816 participants from 11 European countries (Denmark, Sweden, Austria, France, Germany, Switzerland, Belgium, the Netherlands, Spain, Italy and Greece) and Israel were recruited. Since then, participants have been interviewed biennially. The Survey of Health, Ageing and Retirement in Europe has been reviewed and approved by the Ethics Committee of the University of Mannheim [20].

### Wellbeing

Wellbeing at wave 1 was assessed with the CASP-12—an abridged version of the CASP-19 quality of life questionnaire [21]. The CASP-19 has previously been used to assess wellbeing in a cross-sectional survey of people aged ≥50 with various chronic diseases including arthritis [22]. The CASP-12 consists of 12 statements that the respondent could use to describe their lives (e.g. ‘I feel that my life has meaning’). Participants are asked to indicate how often they feel these statements apply to them by selecting from the response options of ‘often’, ‘sometimes’, ‘rarely’ or ‘never’ (see supplementary documents for further details regarding this scale). Possible scores range from 0 to 48 with higher scores indicating higher levels of wellbeing. For the study sample, internal consistency for the CASP-12 was high (*α* = 0.82).

### Arthritis

In waves 1, 2, 4 and 5, participants were asked whether a doctor had ever told them that they had ‘arthritis including osteoarthritis, or rheumatism’. As has been done previously with the Survey of Health, Ageing and Retirement in Europe [23], participants that did not report a diagnosis at wave 1 but reported a diagnosis in a subsequent wave were classified as incident cases. As data on date of diagnosis was not collected, for the purposes of statistical analysis, the date of the interview (month and year) at which the participant first reported a diagnosis of arthritis was taken as the date of diagnosis.

### Confounding Variables

We chose age; sex; depressive symptoms; socio-economic status—as indexed by real household assets net of any debt—level of education; and prevalent hypertension, diabetes, heart attack and stroke at wave 1 as potential confounders of the relationship between wellbeing and later arthritis incidence. Age, sex, socio-economic status and depressive symptoms have previously been associated with arthritis risk [3, 6, 24–26]; these factors have also been related to levels of subjective wellbeing [27–31]. Hypertension, diabetes, heart attack and stroke commonly co-occur with arthritis (in particular osteoarthritis) [32] and have been associated with lower wellbeing [22].

### Mediating Variables

We chose health behaviours (physical activity, alcohol consumption and smoking status) and body mass index (BMI) as potential mediators of the relationship between wellbeing and later arthritis incidence. These factors have previously been associated with arthritis risk [3, 26, 33, 34] and levels of subjective wellbeing [30, 31, 35, 36].

Participants reported the frequency with which they engaged in vigorous and or moderate physical activity. There were four response options: ‘more than once a week’, ‘once a week’, ‘one to three times a month’, and ‘hardly ever or never’. Responses were dichotomised based on activity frequency—either once a week (or more) or less than once a week. Responses were then summed to create three categories: physical inactivity, moderate but not vigorous activity at least once a week and vigorous physical activity at least once a week. Participants reported their frequency of alcohol consumption; there were four response options: ‘5 days a week or more’, ‘1 to 4 days a week’, ‘twice a month or less’ and ‘not at all’. Participants reported their smoking status (non-smoker, former smoker or current smoker). The EURO-D was used to assess symptoms of depression [37]. The scale consists of 12 items—all of which are taken from the Geriatric Mental State [38]. Socio-economic status was indexed by total household assets, gross value of home, value of any other real estate, value of any share of business and value of any vehicles minus mortgage of main residence. The study sample was divided into quintiles according to total household wealth. Level of education was classified according to the International Standard Classification of Education (ISCED-97) framework; participants were categorised according to their highest level of education: pre-primary or primary, lower secondary, upper or post-secondary and first- or second-stage tertiary. BMI (kg/m^2^) was derived from participant self-reported height and weight. Participants were categorised as underweight (below 18.5 kg/m^2^), normal weight (18.5–24.9 kg/m^2^), overweight (25–29.9 kg/m^2^) and obese (30 kg/m^2^ or above). In order to assess the multicollinearity of the covariates, we ran collinearity diagnostics in a linear regression model entering the covariates as predictors and event time as the dependent variable. Values for tolerance were all >0.1; and values for the variance inflation factor were all <10. Consequently, we included all the covariates in our survival model [39].

### Analytical Sample

Of the 30, 816 people taking part in wave 1, 10,530 were included in the analysis. Participants were excluded if at wave 1 they reported a diagnosis of arthritis or did not know whether they had been diagnosed with arthritis or if they refused to respond to the question (*n* = 5694; 18 %), they only participated in wave 1 (*n* = 6500; 26 %) or they had missing data for wellbeing (*n* = 6661; 36 %). Participants were further excluded if they had missing data on any of the covariate variables (*n* = 1431; 12 %).

Participants excluded due to missing CASP-12 data were older; were more likely to be female; were less physically active; consumed less alcohol; were less likely to smoke, had fewer years of education; had higher depression score; had lower SES; and were more likely to report a history or diabetes, stroke and heart attack (see supplementary table). In order to test for possible bias due to missing data, we used multiple multivariate imputation to impute values of covariates with missing values using IBM SPSS Statistics 21 software. This approach assumes that data is missing at random, that is, the pattern of missingness is systematic and can be predicted by observed data [40]. We assumed data was missing at random as missingness was significantly correlated with other measured variables [40]. The imputation models included survival time, arthritis incidence and the covariate variables. Missing data was imputed for the sample of participants that took part at wave 1, did not report a diagnosis of arthritis at wave 1 and had information on incident arthritis (*n* = 25,122). We generated 35 imputed datasets using chained equation imputation. The pooled effect size from analysis with imputed information was very similar to that obtained from analysis employing the sample with complete data. Consequently, we present results from analysis with complete data here.

### Statistical Analysis

Cox proportional hazards regression was conducted in order to examine the association between CASP-12 scores at baseline and incidence of arthritis over the follow-up period. On the basis of Schoenfeld residuals, we found no evidence that the proportional hazards assumption was violated (all *p* values >0.1). Survival time in days was calculated from the date of the wave 1 interview to the date of first arthritis diagnosis or, for those who did not report such a diagnosis during follow-up, the date of the last follow-up interview. Survival times for those who were diagnosed with arthritis were considered censored at the date of the interview when they first reported this diagnosis. Survival times for those who were not diagnosed with arthritis were censored at the date of their last follow-up interview. Preliminary analysis indicated that the relationship between CASP-12 scores and arthritis incidence did not differ according to sex (*p* for interaction term = 0.768). However, the relationship did differ by age (*p* = 0.002). Consequently, separate Cox proportional hazards regression analysis was conducted for two age groups: <65 and ≥65 (we chose 65 as it represents a traditional cutoff point for ‘older adults’ [41]). We employed three adjustment models, in which we first adjusted for potential confounding factors (models 1–2) and then in addition for potential mediating factors (model 3). Model 1 adjusted for sex; model 2 additionally adjusted for depressive symptom score, total household wealth, education and prevalent hypertension, diabetes, heart attack and stroke at wave 1; and model 3 additionally adjusted for health behaviours and BMI.

In order to reduce the risk of reverse causality (i.e. undiagnosed pre-existing arthritis influencing wellbeing), the regression was repeated excluding the first 2 years of follow-up. Hazard ratios (HR) and 95 % confidence intervals (CI) are expressed according to a standard deviation (SD) increase in CASP-12 score.

Finally, we carried out an additional analysis in which we used the Bayes information criterion (a measure of model fit [42]) to test whether a model predicting incident arthritis that contained age, sex, depressive symptoms and CASP-12 scores fitted the data better than the same model without CASP-12 scores.

## Results

Table 1 shows the baseline characteristics of the sample (*n =* 10,530) according to tertiles of wellbeing. On average, people with higher wellbeing were younger; had lower depressive symptom scores; were wealthier; had a lower BMI; were more physically active; consumed more alcohol; had a higher level of education; and were less likely to report a history of hypertension, diabetes, stroke or heart attack than people reporting low levels of wellbeing.

**Table 1:** Baseline characteristics stratified according to tertiles of CASP-12 scores (lowest, middle and highest subjective wellbeing), total *n* = 10,530

Characteristics	Lowest tertile	Middle tertile	Highest tertile	*p* trend
Age (years), mean (SD)	63.92 (10.38)	62.51 (9.64)	61.88 (8.91)	<0.001
Depressive symptoms, Mdn (IQR)	2 (1–4)	1 (1–3)	1 (0–2)	<0.001
Net assets (€), Mdn (IQR)	123,675.50	194,800.00	246,590.00	<0.001
	(30,542.00–257,790.50)	(69,000.00–451,166.00)	(92,969.00–678,260.00)	
Female, no. (%)	1946 (52.82)	1757 (50.50)	1737 (51.59)	0.15
BMI (kg/m^2^), mean (SD)	26.70 (4.28)	26.19 (4.09)	25.84 (3.83)	<0.001
Physical activity, no. (%)				<0.001
Physically inactive	535 (14.52)	219 (6.29)	163 (4.84)	
Moderate physical activity	1368 (37.13)	1238 (35.58)	1045 (31.04)	
Vigorous physical activity	1781 (48.34)	2022 (58.12)	2159 (64.12)	
Alcohol consumption, no. (%)				<0.001
5 days a week or more	913 (24.78)	876 (25.18)	953 (28.30)	
1 to 4 days a week	847 (22.99)	1146 (32.94)	1176 (34.93)	
Twice a month or less	795 (21.58)	770 (22.13)	640 (19.01)	
Not at all	1129 (30.65)	687 (19.75)	598 (17.76)	
Smoking status, no. (%)				0.35
Smoker	809 (21.96)	713 (20.49)	638 (18.95)	
Former smoker	1008 (27.36)	1067 (30.67)	1064 (31.60)	
Non-smoker	1867 (50.68)	1699 (48.84)	1665 (49.45)	
Education, no. (%)				<0.001
Pre-primary or primary	1437 (39.01)	839 (24.12)	592 (17.58)	
Lower secondary	716 (19.44)	625 (17.96)	651 (19.33)	
Upper or post-secondary	1044 (28.34)	1175 (33.77)	1156 (34.33)	
First- or second-stage tertiary	487 (13.22)	840 (24.14)	1156 (34.33)	
History of hypertension	1250 (33.93)	1009 (29.00)	868 (25.78)	<0.001
History of diabetes	366 (9.93)	257 (7.39)	225 (6.68)	<0.001
History of stroke	162 (4.40)	95 (2.73)	61 (1.81)	<0.001
History of heart attack	497 (13.49)	367 (10.55)	231 (6.86)	<0.001

*Mdn* median, *IQR* interquartile ratio

Table 2 displays the HRs for incident arthritis in the two age groups according to a SD increase in overall CASP-12 score.

**Table 2:** Hazard ratios (95 % confidence intervals) for incident arthritis according to a standard deviation increase in CASP-12 stratified by age (under 65 and 65 and over)

Age group	Cases/number	Model 1^a^	Model 2^b^	Model 3^c^
		HR (95 % CI)	HR (95 % CI)	HR (95 % CI)
<65	1240/6476	0.73 (0.69–0.77)**	0.81 (0.76–0.87)**	0.82 (0.77–0.87)**
≥65	1053/4054	0.80 (0.77–0.85)**	0.87 (0.81–0.93)**	0.88 (0.82–0.95)**

*HR* hazard ratio

**p < 0.001

^a^Adjusted for sex

^b^Further adjusted for total net assets (divided into quintiles); EURO-D score; ISCED-97 coding of education (pre-primary or primary, lower secondary, upper secondary or post-secondary, first- or second-stage tertiary); and history of hypertension, diabetes (type 1 and 2), stroke and heart attack

^c^Further adjusted for smoking (never, former, current); alcohol consumption (5 days a week or more, 1 to 4 days a week, twice a month or less, not at all); physical activity (inactive, moderate activity at least once a week, vigorous activity at least once a week); and BMI category (below 18.5; 18.5–24.9; 25–29.9; 30 and above)

There were 2293 reported cases of incident arthritis between waves 2 and 5 (Table 2). Incidence was greater in the older age group—with 19 % incidence in the under 65 age group and 26 % incidence in the 65 and over age group. People with higher CASP-12 scores had a significantly lower risk of incident arthritis after adjustment for sex. This reduction in risk was higher for the younger age group. A SD increase in CASP-12 score was associated with a 27 % (HR 0.73; 95 % CI 0.69–0.77) decrease in arthritis risk in the under 65 age group and a 20 % (HR 0.80; 95 % CI 0.77–0.85) decrease in arthritis risk in the 65 and over age group. The association between CASP-12 scores and reduced arthritis risk was attenuated by adjusting for the additional confounding covariates in model 2 (depressive symptoms, wealth, education and comorbidities). A SD increase in CASP-12 score was associated with a 19 % (HR 0.81; 95 % CI 0.76–0.87) decrease in arthritis risk in the under 65 age group and a 13 % (HR 0.87; 95 % CI 0.81–0.93) decrease in arthritis risk in the 65 and over age group. The risk association was only slightly attenuated by additionally adjusting for potential mediating variables (health behaviours and BMI) in model 3. In the fully adjusted model, a SD increase in CASP-12 score was associated with an 18 % (HR 0.82; 95 % CI 0.77–0.87) decrease in arthritis risk in the under 65 age group and a 12 % (HR 0.88; 95 % CI 0.82–0.95) decrease in arthritis risk in the 65 and over age group.

In addition to CASP-12 score, significant predictors of reduced arthritis risk in both age groups included being male and lower depressive symptom score. Additional predictors of reduced risk in the <65 age group were higher educational attainment, having a BMI above 18.5 or below 30 and no history of hypertension. Additional predictors of reduced risk in the ≥65 age group were greater wealth, greater alcohol consumption, not smoking and having a BMI lower than 30.

The observed association between higher CASP-12 score and reduced arthritis risk was little changed by the exclusion of cases diagnosed in the first 2 years of follow-up or the exclusion of participants who had not reported a diagnosis of arthritis at baseline but who did say that they were bothered by back, knee, hip or joint pain at that time.

Analysis assessing model fit revealed a better fit for the model including CASP-12 score, depressive symptoms, age and sex (Bayes information criterion 42,354.75) compared with the same model excluding CASP-12 score (Bayes information criterion 42,402.88) (a lower Bayes information criterion score indicates a better model fit); this difference was highly significant (*p* < 0.0001).

## Discussion

In a sample representative of people aged 50 or over living in Europe and Israel, higher levels of wellbeing at baseline were associated with a reduced risk of incident arthritis over a 9-year follow-up period. This association remained significant although attenuated after adjusting for established risk factors. The strength of the association between higher wellbeing and reduced arthritis risk was dependent on age—with a stronger association found in the younger age group.

This is the second study to demonstrate an association between higher wellbeing and a reduced risk of incident arthritis. The current results demonstrate that our previous findings regarding the association between higher wellbeing and reduced arthritis risk in an English sample of older adults [18] can be replicated in a cross-national sample. More generally, our results support the theory that psychosocial factors may be implicated in the aetiology of arthritis.

The association between higher wellbeing and reduced arthritis risk appears to be partially confounded by depressive symptoms, SES and comorbidities as adjusting for these variables attenuated the association between wellbeing and arthritis risk. Despite this attenuation, HRs remained significant, suggesting that the association between higher wellbeing and reduced arthritis risk is, in part, independent of these factors. This finding is consistent with previous studies that documented a significant association between wellbeing and disease risk after controlling for depressive symptoms and established risk factors [16, 17, 43]. However, these previous studies did not report whether adding wellbeing to a model containing depressive symptoms increased its ability to predict disease incidence. We tested this in our analysis; we found that a model containing CASP-12 scores and depressive symptoms was a significantly better fit to the data than the model excluding CASP-12 scores. These results support the idea that wellbeing and depressive symptoms are independently associated with arthritis risk.

We identified health behaviours and BMI as potential mediators of this association; however, additionally adjusting for these variables in model 3 only attenuated the association slightly (by 5 % for the <65 age group and by 8 % for the ≥65 age group). HRs remained significant in the fully adjusted model, suggesting that health behaviours and BMI explain only a small part of the relationship between wellbeing and incident arthritis. Additional factors may underlie this association.

A possible explanation for the association between greater wellbeing and reduced arthritis risk is that wellbeing may impact directly on physiological processes relevant to arthritis risk or symptom expression. Higher wellbeing is associated with a reduced inflammatory response [44–46]. Thus, higher wellbeing could lessen the expression of arthritic symptoms, by reducing the extent of inflammation. Alternatively, wellbeing may impact on the experience rather than the expression of arthritis pain. Pain is a complex phenomenon that can be influenced by multiple factors including positive affect. High positive state and trait affect is associated with reduced pain perception [47, 48]; thus, high wellbeing may reduce the experience of arthritis pain. Both mechanisms (reduced inflammation or pain perception) could reduce the likelihood of an individual with high wellbeing from seeking a formal diagnosis of arthritis.

Although the association between higher wellbeing and reduced arthritis risk remained significant in the fully adjusted model for both age groups, our results suggests a stronger association for those younger than 65. Arnold et al. [49] have found that onset of arthritis at older ages is more frequently associated with higher disease activity and disability compared with arthritis onset at younger ages. The weaker effect observed for the older age group could reflect the fact that wellbeing is less potentially protective against the onset of these more severe forms of arthritis.

### Study Limitations

Our study has a number of strengths, including its size, the fact that the cohort was designed to be representative of people aged 50 and over in Europe and Israel, and the availability of data on a range of potential confounding and mediating factors. Our study also has some limitations. Firstly, information on arthritis diagnosis by a doctor was based on self-reports. This may have affected the accuracy of the arthritis incidence variable, although self-report of arthritis diagnosis is generally consistent with clinically derived diagnoses [50]. Second, reverse causality needs to be considered. Research suggests that there is a delay between the onset of symptoms and time of arthritis diagnosis [51]. However, analysis firstly excluding participants diagnosed within the first 2 years of follow-up and secondly excluding participants who had not reported a diagnosis of arthritis at baseline but who said they were bothered by back, knee, hip or joint pain at that time led to a minimal change in HRs. This suggests that the association between higher wellbeing and reduced incident arthritis is unlikely to be due to the experience of arthritis symptoms prior to diagnosis. Third, we were unable to control for hyperuricemia, which is associated with increased risk of osteoarthritis and may be associated with reduced quality of life. Although we controlled for depressive symptoms, we were unable to control for other negative psychosocial factors previously associated with arthritis risk including perceived stress [6] and psychological trauma [11]. Fourth, a substantial proportion of participants were excluded from the analysis due to missing wellbeing data. This may have introduced a source of bias as excluded participants differed from included participants on a number of covariates (see supplementary table). However, analysis with imputed missing covariate data yielded similar effect sizes to those obtained for the sample with complete data suggesting that this exclusion did not significantly bias our results. Finally, we were unable to distinguish between rheumatoid arthritis and osteoarthritis in the analysis as separate data on these conditions was only collected in the most recent wave of data used here. Osteoarthritis is a degenerative joint disease caused by a breakdown of cartilage; rheumatoid arthritis, on the other hand, is defined as an autoimmune inflammatory disorder. Considering this difference in pathophysiology, it is likely that the nature of the association between higher wellbeing and reduced arthritis risk is qualitatively different for the two conditions. Few studies into the association between psychosocial factors and arthritis have distinguished between these two conditions. One previous study into the association between diagnosis of arthritis and subsequent onset of psychiatric disorders found a stronger association for osteoarthritis compared with rheumatoid arthritis. However, this study identified arthritis as a predictor rather than a consequence of psychiatric disorders. The prevalence of osteoarthritis is significantly higher than that of rheumatoid arthritis particularly in those aged over 50 [52]. Data available from wave 5 of the Survey of Health, Ageing and Retirement in Europe suggest that this was also the case for our sample; at wave 5 (the first wave at which data were collected on arthritis type), 9 % of the sample reported a diagnosis of rheumatoid arthritis and 18 % reported a diagnosis of osteoarthritis. Thus, it is likely that the association between wellbeing and arthritis incidence observed in the current study predominantly reflects the association between wellbeing and osteoarthritis incidence. Further follow-up of the cohort will be needed to confirm that.

## Conclusions

Our study provides further evidence for an association between higher wellbeing and reduced arthritis risk. This association appears to be confounded by SES, education, comorbidities and depressive symptoms and in part mediated by health behaviours and BMI; however, additional factors including immune processes and pain perception could play a role. There is evidence that public interventions can effectively increase levels of wellbeing, particularly at older ages [53–55]. Our findings suggest that interventions to improve levels of wellbeing may help reduce the incidence of arthritis.

## Electronic Supplementary Material

Below is the link to the electronic supplementary material.

ESM 1 (DOCX 16 kb)

ESM 2 (DOCX 18.4 kb)
