# Supplementary material for: Wellbeing and Arthritis Incidence: the Survey of Health, Ageing and Retirement in Europe
Source: Ann Behav Med. 2016 Jan 14;50:419–26. doi: 10.1007/s12160-015-9764-6 (PMC4869763; doi:10.1007/s12160-015-9764-6)
Supplement: Supplementary file 1 — (DOCX 16 kb) [file 12160_2015_9764_MOESM1_ESM.docx]

| **Here is a list of statements that people have used to describe their lives or how they feel. We would like to know how often, if at all, you think this applies to you.** (Please tick one box in each row) | | | | | |
| --- | --- | --- | --- | --- | --- |
|  |  | Often_1_ | Sometimes_1_ | Rarely_1_ | Never_1_ |
|  |  | ▼_1_ | ▼_1_ | ▼_1_ | ▼_1_ |
| a) | My age prevents me from doing the things I would like to | 🞏_1_ | 🞏_2_ | 🞏_3_ | 🞏_4_ |
| b) | I feel that what happens to me is out of my control | 🞏_1_ | 🞏_2_ | 🞏_3_ | 🞏_4_ |
| c) | I feel left out of things | 🞏_1_ | 🞏_2_ | 🞏_3_ | 🞏_4_ |
| d) | I can do the things that I want to do | 🞏_1_ | 🞏_2_ | 🞏_3_ | 🞏_4_ |
| e) | Family responsibilities prevent me from doing what I want to do | 🞏_1_ | 🞏_2_ | 🞏_3_ | 🞏_4_ |
| f) | Shortage of money stops me from doing the things I want to do | 🞏_1_ | 🞏_2_ | 🞏_3_ | 🞏_4_ |
| g) | I look forward to each day | 🞏_1_ | 🞏_2_ | 🞏_3_ | 🞏_4_ |
| h) | I feel that my life has meaning | 🞏_1_ | 🞏_2_ | 🞏_3_ | 🞏_4_ |
| i) | On balance, I look back on my life with a sense of happiness | 🞏_1_ | 🞏_2_ | 🞏_3_ | 🞏_4_ |
| j) | I feel full of energy these days | 🞏_1_ | 🞏_2_ | 🞏_3_ | 🞏_4_ |
| k) | I feel that life is full of opportunities | 🞏_1_ | 🞏_2_ | 🞏_3_ | 🞏_4_ |
| l) | I feel that the future looks good for me | 🞏_1_ | 🞏_2_ | 🞏_3_ | 🞏_4_ |
|  |  | ▲_1_ | ▲_1_ | ▲_1_ | ▲_1_ |
|  |  | Often_1_ | Sometimes_1_ | Rarely_1_ | Never_1_ |

Supplementary Material: CASP-12 questionnaire
