# Supplementary material for: Wellbeing and Arthritis Incidence: the Survey of Health, Ageing and Retirement in Europe
Source: Ann Behav Med. 2016 Jan 14;50:419–26. doi: 10.1007/s12160-015-9764-6 (PMC4869763; doi:10.1007/s12160-015-9764-6)
Supplement: Supplementary file 2 — (DOCX 18.4 kb) [file 12160_2015_9764_MOESM2_ESM.docx]

| **Supplementary Table:** Baseline characteristics for participants with complete and missing  CASP-12 data | | | | | |
| --- | --- | --- | --- | --- | --- |
| Characteristics | Complete | N for complete^a^ | Missing | N for missing^a^ | *p-*trend |
| Age (yrs), Mean (SD) | 63.64 (10.13) | 18470 | 65.12 (11.13) | 12033 | <0.001 |
| Female, No. (%) | 10157 (55.00) | 18470 | 6805 (56.60) | 12033 | 0.007 |
| Physical Activity, No. (%) |  | 18664 |  | 11971 | <0.001 |
| Physically inactive | 2408 (12.90) |  | 2267 (18.93) |  |  |
| Moderate physical activity | 6503 (34.84) |  | 4180 (34.91) |  |  |
| Vigorous physical activity | 9753 (52.23) |  | 5524 (46.14) |  |  |
| Alcohol consumption, No. (%) |  | 18665 |  | 11976 | <0.001 |
| 5 days a week or more | 4402 (23.58) |  | 2828 (23.61) |  |  |
| 1 to 4 days a week | 4939 (26.46) |  | 2678 (22.36) |  |  |
| Twice a month or less | 3755 (20.12) |  | 2134 (17.82) |  |  |
| Not at all | 5569 (29.84) |  | 4336 (35.21) |  |  |
| Smoking status, No. (%) |  | 18668 |  | 11979 | <0.001 |
| Smoker | 3699 (19.81) |  | 2211 (18.46) |  |  |
| Former smoker | 5315 (28.47) |  | 3169 (26.45) |  |  |
| Non smoker | 9654 (51.71) |  | 6599 (55.09) |  |  |
| Education, No. (%) |  | 18521 |  | 11949 | <0.001 |
| Pre-primary or primary | 5537 (29.90) |  | 4506 (37.71) |  |  |
| Lower secondary, | 3377 (18.23) |  | 2070 (17.32) |  |  |
| Upper or post-secondary | 5901 (32.07) |  | 3335 (27.91) |  |  |
| First or second stage tertiary | 3668 (19.80) |  | 2038 (17.06) |  |  |
| History of hypertension | 5955 (31.91) | 18662 | 3786 (31.60) | 11982 | 0.567 |
| History of diabetes | 1824 (9.77) | 18662 | 1280 (10.68) | 11982 | 0.010 |
| History of stroke | 600 (3.22) | 18662 | 554 (4.62) | 11982 | <0.001 |
| History of heart attack | 2195 (11.76) | 18662 | 1596 (13.32) | 11982 | <0.001 |
| History of arthritis | 3226 (17.27) | 18676 | 2468 (20.34) | 12140 | <0.001 |
| Depressive symptoms, *Mdn* (IQR) | 2 (1-3) | 18476 | 2 (1-4) | 11474 | <0.001 |
| Net Assets (€)  *Mdn* (IQR) | 156080.00-  (41000.00  -365000.00) | 17110 | 150000.00  (25000.00-  330000.50) | 11108 | <0.001 |
| BMI (kg/m^2)^  Mean (SD) | 26.38 (4.28) | 18377 | 26.33 (4.37) | 11731 | 0.315 |

^a^ N with complete and missing CASP-12 data is lower for some covariates due to a higher proportion of missing data on those covariates.
